# Functional and Transcriptome Analysis of *Streptococcus pyogenes* Virulence on Loss of Its Secreted Esterase

**DOI:** 10.3390/ijms23147954

**Published:** 2022-07-19

**Authors:** Xiaolan Zhang, Yue Wang, Hui Zhu, Zhaohua Zhong

**Affiliations:** College of Basic Medical Sciences, Harbin Medical University, Harbin 150081, China; zhangxl@ems.hrbmu.edu.cn (X.Z.); 18204608083@163.com (Y.W.)

**Keywords:** *Streptococcus pyogenes*, esterase, virulence, invasion, infection

## Abstract

Esterases are broadly expressed in bacteria, but much remains unknown about their pathogenic effect. In previous studies, we focused on an esterase secreted by *Streptococcus* *pyogenes* (group A *Streptococcus*, GAS). Streptococcal secreted esterase (Sse) can hydrolyze the sn−2 ester bonds of platelet−activating factor (PAF), converting it to an inactive form that inhibits neutrophil chemotaxis to the infection sites. However, as a virulent protein, Sse probably participates in GAS pathogenesis far beyond chemotaxis inhibition. In this study, we generated the *sse* gene knockout strain (Δ*sse*) from the parent strain MGAS5005 (hypervirulent M1T1 serotype) and compared the difference in phenotypes. Absence of Sse was related to weakened skin invasion in a murine infection model, and significantly reduced GAS epithelial adherence, invasion, and intracellular survival. Reduced virulence of the Δ*sse* mutant strain was explored through transcriptome analysis, revealing a striking reduction in the abundance of invasive virulence factors including M protein, SIC, ScpA, and SclA. Besides the influence on the virulence, Sse also affected carbohydrate, amino acid, pyrimidine, and purine metabolism pathways. By elucidating Sse−mediated pathogenic process, the study will contribute to the development of new therapeutic agents that target bacterial esterases to control clinical GAS infections.

## 1. Introduction

*Streptococcus pyogenes*, also called group A *Streptococcus* (GAS), cause a variety of infection−related diseases, such as pharyngitis, scarlet fever, cellulitis, severe invasive infections, and rheumatic heart disease [1,2,3,4]. Over half a million deaths are due to invasive GAS infections and rheumatic heart disease every year, which indicates that GAS is still a serious threat to global public health [2,4]. GAS produces an arsenal of virulence factors that hydrolyze host substrates, destroy tissue barriers, and resist antimicrobial pathways [4]. One of these virulence factors is streptococcal secreted esterase (Sse), which inhibits neutrophil recruitment to the infection sites by hydrolyzing PAF [5,6]. The hydrolysis function of Sse is similar to that of human PAF acetylhydrolase, but the enzymatic activity is over 30−fold higher than that of the latter [6]. In our previous studies, it was demonstrated that Sse participates in the pathogenesis of multiple GAS serotypes (MGAS5005, MGAS2221, MGAS315, and MGAS6180), particularly the hypervirulent CovS mutant strain MGAS5005 [7,8]. Besides impeding chemotaxis, Sse could enhance the ability of GAS in resisting neutrophil phagocytosis [8]. However, the role of Sse in GAS pathogenesis seems to be not fully understood. As virulence factors do not work in isolation, it will be valuable to determine which virulence factors work together with Sse in the disease process. In this study, to reveal the overall impact of Sse on GAS, functional tests and transcriptome sequencing have been performed. The absence of Sse significantly affects the expression of several invasive virulence factors and GAS invasion to epithelial cells. This study has discovered the role of a bacterial esterase in epithelial invasion.

## 2. Results

### 2.1. Sse Enhances GAS Skin Invasion in a Murine Infection Model

**Figure 1:**
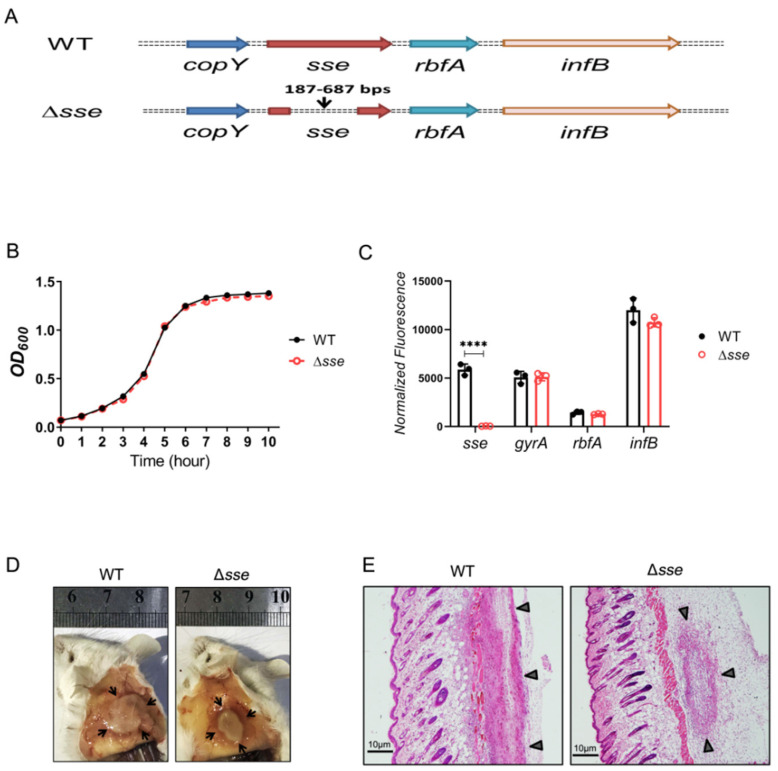
Deletion of the *sse* gene does not affect GAS growth but does reduce skin invasion. (**A**) Schematic of the genomic organization of *sse* loci in MGAS5005 (top) and the Δ*sse* mutant (bottom). The sequence of 187−687 bps is deleted by an in-frame allelic replacement of *sse* from MGAS5005. (**B**) The GAS growth curves in THY medium. (**C**) Quantification of mRNA transcripts of *sse*, *gyrA*, *rbfA*, and *infB*. Data are plotted as the mean ± SD, *n* = 3. Symbols in statistical analyses: **** *p <* 0.0001. (**D**) Representative images of skin lesions in mice infected with MGAS5005 and the Δ*sse* mutant. The abscess is indicated by black arrows. (**E**) Microscopic pictures of the HE-stained skin lesions, which are indicated by black triangles. Scale bar, 10 μm.

We constructed an in−frame allelic exchange mutant (Δ*sse*, the deletion sequence is between 187–687 bps) in the background of the well characterized globally disseminated M1T1 serotype MGAS5005 (Figure 1A). It was necessary to observe the growth of the Δ*sse* mutant strain compared with the wild type (WT) strain in vitro. The growth curve of the mutant strain had an obvious lag phase, logarithmic phase, and stationary phase that were similar to the WT strain (Figure 1B). Thus, the Δ*sse* mutant strain exhibited no growth defect in vitro. The Δ*sse* mutant strain did not exert polar effects on the expression of downstream genes, since the abundance of *rbfA*, *infB*, and the reference gene *gyrA* did not differ significantly between the Δ*sse* mutant strain and the WT strain (Figure 1C). Furthermore, mice were subcutaneously infected with GAS and the skin lesions were observed via HE staining. Mice infected with the Δ*sse* mutant strain exhibited a bounded abscess, which was quite different from the disseminated lesion caused by the WT strain (Figure 1D,E). These results suggest that Sse plays a role in GAS skin invasion.

### 2.2. Sse Significantly Promotes GAS Invasion and Intracellular Survival in the Epithelial Cells

**Figure 2:**
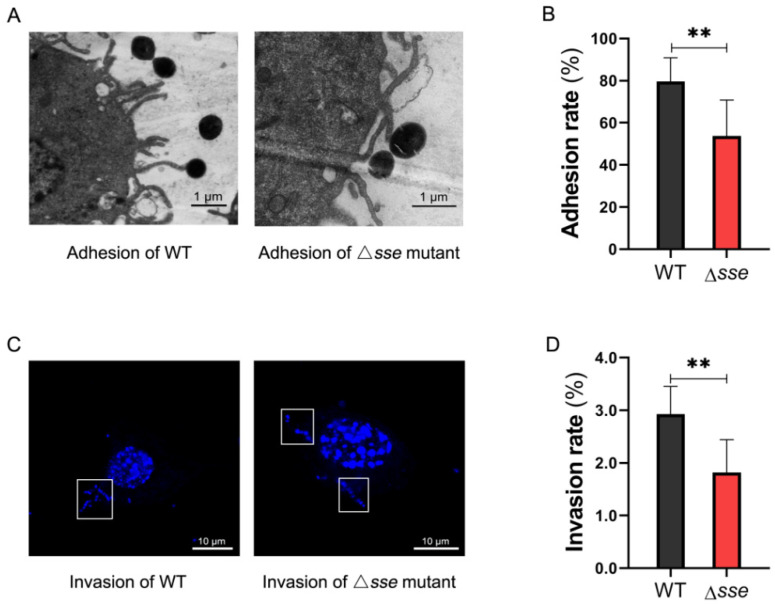
Adhesion and invasion of GAS to Detroit562 cells. (**A**) Capacity of adhesion to Detroit562 cells. Cells were infected with GAS at an MOI of 100 for 4 h. The sections were observed by transmission electron microscopy. Scale bar, 1 μm. (**B**) The adhesion rates were calculated by colony counting, which normalized the GAS counts at 4 h postinfection with that of the initial. Data are represented as the mean ± SD, *n* = 8. ** *p* < 0.01. (**C**) Capacity of invasion to Detroit562 cells. Cells were fixed at 4 h postinfection and stained with DAPI, which was used for cell nucleus and GAS DNA staining. Specimens were examined by laser confocal microscopy (intracellular GAS was inside the white frame). Scale bar, 10 μm. (**D**) The invasion rates were calculated as described above. Gentamicin and penicillin were added to kill extracellular bacteria. Data are represented as the mean ± SD, *n* = 6. ** *p* < 0.01.

**Figure 3:**
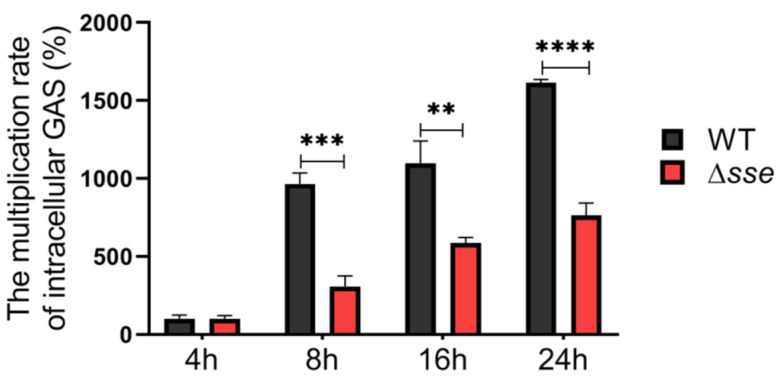
Intracellular multiplication rates of GAS by colony counting. It normalized the intracellular GAS counts at 8, 16, and 24 h with that of 4 h postinfection. Data are plotted as the mean ± SD, *n* = 3. ** *p* < 0.01, *** *p* < 0.001, **** *p* < 0.0001.

GAS adhered to epithelial cells and subsequently invaded and penetrated cells to various degrees (Figure 2A,C). The adhesion rate of the Δ*sse* mutant strain was 53.77%, which was lower than the 79.62% of the WT strain (*p* = 0.003), and its invasion rate was 1.8%, which was also lower than the 2.9% of the WT strain (*p* = 0.0075, Figure 2B,D). Therefore, the presence of Sse significantly promotes GAS epithelial adhesion and invasion. To further explore the difference in intracellular survival, the intracellular GAS multiplication at 8, 16, and 24 h were compared to the intracellular GAS numbers at 4 h postinfection, respectively. GAS can proliferate in the epithelial cells over time; however, the multiplication rates of the Δ*sse* mutant were all significantly lower than those of the WT strain (Figure 3). Therefore, these results demonstrate that Sse markedly promotes the invasion and intracellular survival of GAS in the epithelial cells.

### 2.3. Sse Probably Inhibits Epithelial Phagocytic Function to Promote Intracellular GAS Proliferation

**Figure 4:**
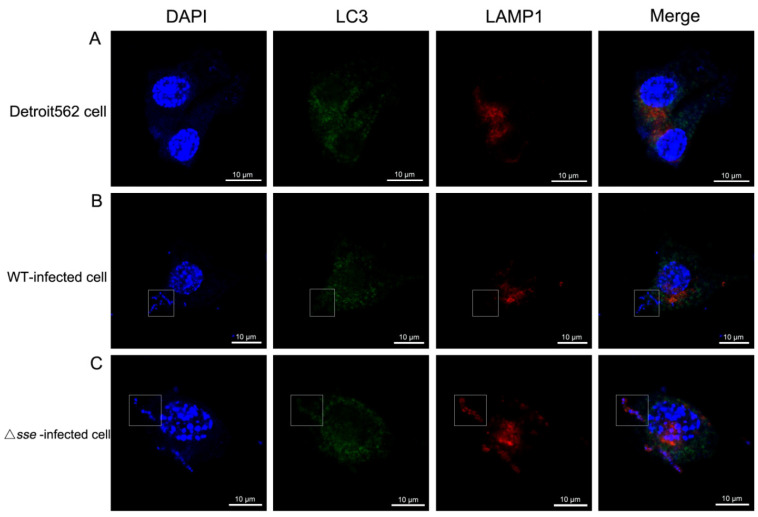
The colocalization of GAS, LC3−positive phagosomes, and LAMP1 positive lysosomes inside the epithelial cells. Cells were fixed at 4 h postinfection and stained with anti−LC3 and anti−LAMP1 antibodies. DAPI was used for cell nucleus and GAS DNA staining. Specimens were observed by laser confocal microscopy (intracellular GAS was inside the white frame). Scale bar, 10 μm. (**A**) Detroit562 cell. (**B**) WT−infected cell. (**C**) The Δ*sse* mutant−infected cell.

Once entering the epithelial cell, GAS would be enwrapped in the phagosomes and then fused with lysosomes. However, it remains unclear whether there are differences in resisting epithelial clearance between the Δ*sse* mutant strain and the WT strain. To address this question, we examined the colocalization of GAS, LC3−positive phagosomes, and lysosomes using immunofluorescence at 4 h postinfection. We found that the number of LC3−surrounded Δ*sse* mutants increased, but the number of LC3−surrounded WT strain decreased distinctly (Figure 4B,C). More lysosomes fused into LC3−positive phagosomes, inside which the Δ*sse* mutant was also present (inside the white frame). This result indicates that Sse probably inhibits epithelial phagocytic function to promote intracellular GAS multiplication.

### 2.4. Sse Increases the Transcription of Invasive Virulence Factors and Affects Multiple Metabolic Pathways

**Figure 5:**
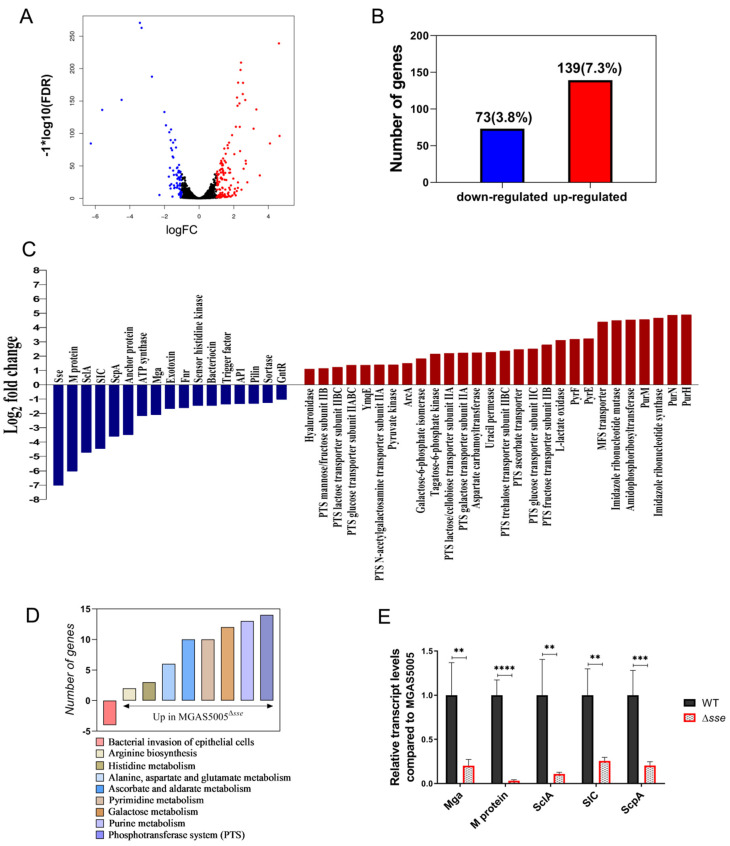
Deletion of the *sse* gene is associated with transcriptome remodeling and reduced abundance of invasive virulence factors. (**A**) The volcano plot of differentially expressed genes. Blue dots represent down−regulated genes and red dots represent up−regulated genes. (**B**) A statistical graph of differentially expressed genes. (**C**) Part of down−regulated genes (blue) and up−regulated genes (red) in the Δ*sse* mutant. Log2 fold change ratio of the mean read count values between the wild type and the Δ*sse* mutant. *n* = 3. (**D**) The number of differentially expressed genes between the wild type and the Δ*sse* mutant through the pathway enrichment in KEGG (Kyoto Encyclopedia of Genes and Genomes). (**E**) The transcript levels of virulence factors identified by RT−PCR. Data are plotted as the mean ± SD. *n* = 6. ** *p* < 0.01, *** *p* < 0.001, **** *p* < 0.0001.

To reveal the overall impact of Sse on GAS, we compared the transcriptional profiles from GAS grown to mid−logarithmic phase. We found that the deletion of *sse* obviously remodeled GAS transcriptome. From a total of 1896 genes identified, the abundance of 73 genes (3.8% of the GAS genome) was decreased, while that of 139 genes was increased in the Δ*sse* mutant strain, compared to the WT strain (Figure 5A,B). The up−regulated genes were associated with multiple metabolic pathways, mainly including carbohydrate, amino acid, pyrimidine, and purine metabolism pathways (Figure 5C,D). On the other hand, the down−regulated genes were several important virulence factors and regulators of GAS. Four of these down−regulated virulence factors, including M protein, streptococcal inhibitor of complement (SIC), streptococcal C5a peptidase (ScpA), streptococcal collagen−like protein (SclA) were involved in bacterial invasion of epithelial cell (Figure 5C,D). Furthermore, the transcriptional levels of four virulence factors and their regulator Mga were identified by RT−PCR (Figure 5E). These results indicate that Sse has regulatory effects on multiple metabolism pathways and probably promotes GAS invasion by working together with invasive virulence factors.

## 3. Discussion

Bacterial pathogens can escape host immune clearance and extracellular antibiotics once they invade human cells [9,10,11,12]. This often causes persistence and enables recurrent infections, such as recurrent streptococcal tonsillitis in children [13,14]. In this study, we have first discovered an esterase (Sse) secreted by GAS that significantly promotes the invasion and survival in human epithelial cells. The Δ*sse* mutant strain showed significantly reduced internalization and intracellular multiplication into epithelial cells. Moreover, LC3−associated phagocytosis is recognized as an important antimicrobial pathway for epithelial cells [15,16]. There was significant influence on that phagocytic pathway in the absence of Sse, which was consistent with the results of colony counting. We speculate that Sse may mediate GAS invasion and inhibit phagocytosis in epithelial cells.

**Figure 6:**
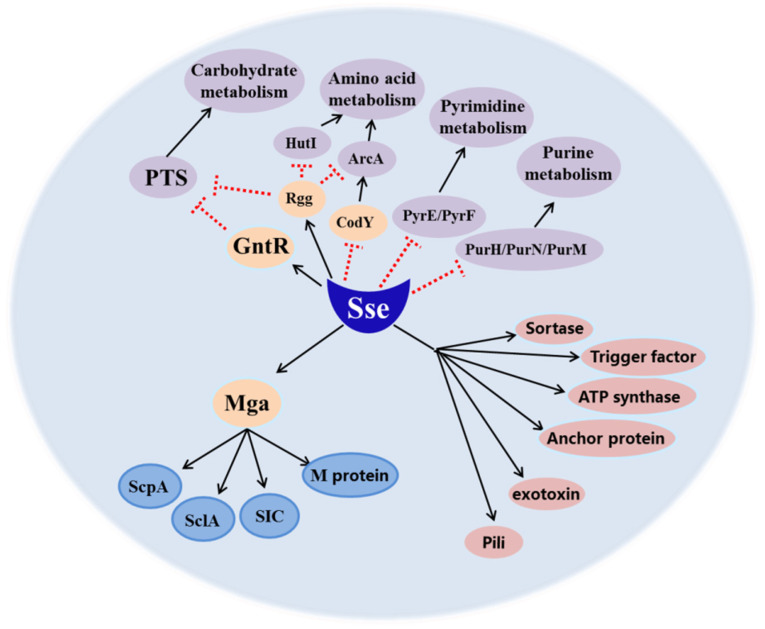
Schematic representation of the network of GAS virulence and metabolism affected by Sse. Sse may activate (black arrows) expression of invasive virulence factors (M protein, SIC, SclA, ScpA) through the regulator Mga and other virulence determinants (highlighted in pink). Conversely, Sse negatively controls (dashed red lines) the expression of factors in multiple metabolic pathways. Consequently, Sse is widely involved in GAS pathogenesis.

To elucidate the effects of Sse on the transcriptome of MGAS5005, we compared the transcriptional profiles of the Δ*sse* mutant grown to mid-logarithmic phase with the wild−type strain. Based on our results, we drew a schematic diagram for Sse−mediated virulence and metabolism in GAS (Figure 6), in which Sse could affect the expression of GAS proteins both directly or indirectly through regulators. After the analysis, four down−regulated virulence factors controlled by the regulator Mga have been found, including M protein, SIC, ScpA, and SclA [17], and they are all involved in bacterial invasion of epithelial cell. These virulence factors probably contribute directly or indirectly to the invasion process of actin rearrangement or endocytic pathways, which ultimately lead to the internalization of GAS [18,19,20,21]. M protein is a major surface virulence factor that binds to a wide range of host cells and proteins, including plasminogen, IgA, IgG, factor H, and C4b-binding protein [22,23]. It can efficiently promote uptake of streptococci by both human endothelial and epithelial cells. SICis also a secreted virulence factor that inhibits complement-mediated lysis and contributes to resist lysozyme, LL−37, and defensins [18,24]. ScpA is a surface serine protease that cleaves the complement component C5a binding sites to interfere with neutrophil recruitment [19,25]. SclAcan bind to pharyngeal and fibroblast cells reacting with α_2_β_1_ integrin through collagen [20,21]. Taken together, these results indicate that the reduced invasion and intracellular multiplication of the Δ*sse* mutant to epithelial cells are probably mediated by the loss of Sse and these virulence factors. According to our studies, in the absence of Sse, GAS invasion is significantly attenuated. We have speculated that there are three main reasons for this. First, Sse can hydrolyze the sn−2 ester bonds of PAF, converting it to an inactive form that inhibits neutrophil chemotaxis to the infection sites [5]. Second, Sse contributes to resisting phagocytosis by neutrophils at the early stage of GAS infection [8]. Third, Sse promotes the transcription of invasive virulence factors including M protein, SIC, ScpA, and SclA. Thus, Sse is involved in GAS invasion of the host.

The absence of Sse also down-regulates the expression of sortase, trigger factor, pili, etc., which attenuates the virulence of the Δ*sse* mutant. Besides its role in virulence, Sse has regulatory effects on carbohydrate, amino acid, pyrimidine, and purine metabolism pathways. The absence of Sse leads to enhanced transcripts of genes which transport, deal with, and regulate non−glucose sugars (fructose, mannose, sucrose), arginine, and histidine. Those may be important alternative ways to compensate for the lack of energy sources in the Δ*sse* mutant. In this manner, Sse could interact with the operator regions of genes observed in our study, leading to alteration of the corresponding transcripts. Further studies are needed to confirm this hypothesis or other potential mechanisms of the Sse-mediated pathogenic process, which can elucidate the overall roles of a bacterial esterase in GAS pathogenesis.

In this study, it was found that a bacterial esterase Sse promotes GAS invasion and intracellular survival through enhancing the transcription of invasive virulence factors and affecting specific metabolic pathways of GAS.

## 4. Materials and Methods

### 4.1. Strains and Growth Conditions

The hypervirulent M1T1 serotype MGAS5005 was separated form clinical patients as described previously [7,8]. Its ∆*sse* mutant strain carrying an in−frame allelic replacement of *sse* was also generated as described previously [8]. The ∆*sse* mutant was verified by polymerase chain reaction and sequencing (Life Technologies, Shanghai, China). The deletion of *sse* did not disrupt the open reading frame.

GAS was cultured in Todd−Hewitt broth (BD Bioscience) supplemented with 0.2% (*w*/*v*) yeast extract (Amresco) at 37 °C and 5% CO_2_. Strains were grown to logarithmic phase in THY (OD_600_ ≈ 0.8), pelleted, washed, and resuspended in phosphate-buffered saline (PBS). When plotting the growth curves, the strains were cultured from the same initial quantity (OD_600_ = 0.05).

### 4.2. Mouse Subcutaneous Infection

CD1 mice (18−22 g) were subcutaneously inoculated with 0.2 mL (≈2.0 × 10^8^ CFU) of a bacterial suspension in PBS (*n* = 6/group). The surviving mice were euthanized 2 days after inoculation and the skin lesions were photographed together with the dead mice. Those skin lesion sites were also excised for histopathological analysis. The skin samples were then dehydrated with ethanol, cleared with xylene, and embedded in paraffin. The 5 μm paraffin sections were stained with hematoxylin and eosin (HE staining) according to standard staining procedures. All the protocols were approved by the Institutional Research Board of Harbin Medical University (HMUIRB20190015).

### 4.3. Adhesion and Invasion Assays

Human nasopharyngeal epithelial cells (Detroit562) were grown in minimum essential medium (HyClone) supplemented with 10% fetal bovine serum (Genial) at 37 °C and 5% CO_2_. The confluent monolayer was infected with MGAS5005 or the ∆sse mutant using a multiplicity of infection (MOI) of 100 GAS per cell. After 4 h incubation, infected cells were washed three times with cold PBS and scratched down with sterile distilled water. Those cells were lysed by freezing and thawing thrice. The total colony forming units (CFUs) of GAS were determined by plating the cell lysate at appropriate dilutions on THY agar plates. To calculate the CFUs of invasion, the extracellular GAS was killed by 100 μg/mL gentamicin and 100 μg/mL penicillin one hour ahead of time. The total CFUs minus the CFUs of invasion is equal to the CFUs of adhesion. The adhesion and invasion rates were given in comparison with the initial CFUs at 0 h postinfection. According to above methods, the intracellular multiplication rates of GAS at 8, 16, and 24 h were given in comparison with the intracellular CFUs at 4 h postinfection.

### 4.4. RNA Extraction and Transcriptome Sequencing

MGAS5005 and the ∆*sse* mutant were grown to logarithmic phase (OD_600_ ≈ 0.8). Total RNA was extracted from GAS with trizol reagent (Ambion). The RNA samples were dissolved in diethylpyrocarbonate (DEPC) and stored at −80 °C. The transcriptome sequencing was based on the Illumina HiSeq (Genewiz life sciences company, Suzhou, China). To find differentially expressed genes, the standard was that the read count values of genes changed more than 2−fold and corrected *p* values were less than 0.05.

### 4.5. Transmission Electron Microscopy

Detroit562 cells were infected with GAS for 4 h and gently collected by trypsinization and centrifugation and resuspended in 2.5% glutaraldehyde at 4 °C. The embedded specimens were cut into ultrathin sections, followed by staining with uranyl acetate and lead citrate solution. The accelerating voltage was 80 kV and the sections were observed under the microscopy (Hitachi7500, Hitachi, Tokyo, Japan).

### 4.6. RT−PCR

Total RNA was prepared from MGAS5005 and the ∆*sse* mutant using trizol reagent and the concentration was adjusted to 100 ng/mL. Sets of primers are listed in Table 1. The RT−PCR amplifications were performed with TB Green Premix Ex TaqⅡkit (Tli RNaseH Plus) (TaKaRa) and measured by an Applied Biosystems 7500. All data were normalized to an internal standard DNA gyrase subunit A (*gyrA*). The transcript levels were calculated relative to the gene expression of MGAS5005.

### 4.7. Immunofluorescence Staining

Detroit562 cells were seeded on glass coverslips (diameter 12 mm) in wells of 24−well culture plates and infected with GAS for 4 h as described above. Cells were fixed with 4% paraformaldehyde, permeabilized with PBS containing 0.1% Triton X−100, blocked with 1% BSA for 1 h, and then incubated with LC3B mouse mAb (83506, Cell Signaling Technology, Danvers, Massachusetts, USA), or LAMP1 rabbit mAb (9091, Cell Signaling Technology) at 4 °C overnight. Next morning, the cells were washed with 0.1% BSA and incubated with Alexafluor 488−conjugated or Alexafluor 555−conjugated IgG (1:1000 dilution, Cell Signaling Technology). Nuclei were stained with DAPI (ZSGB−BIO, Beijing, China). Finally, cells were observed under a laser confocal microscopy (Leica TCS SP8, Wetzlar, Germany).

### 4.8. Statistical Analysis

The data were analyzed by Student’s *t*−test. For the results of the transcriptome, the standard was that the mean read count values of genes changed more than 2−fold and corrected *p* values were less than 0.05. All the figures were generated using GraphPad Prism version 8.0. The *p* value < 0.05 was considered statistically significant.

## Figures and Tables

**Table 1:** Sets of primers of RT−PCR.

Target	Forward Primer **(5′ to 3′)**	Reverse Primer **(5′ to 3′)**
Mga	TACAAGGGACACTCTGCCGTCTAC	GAGGTGAAACAAGCGTGAAAGGTC
M protein	AGGTAAAGGTCAAGCACCACAAGC	GGCTGCCGCTGTGAAGAATGG
SclA	CGCAATGGCAATATGGCTAAG	CTACTGCTGCTGCTGTAAAGA
SIC	GGAGCATTAGGTACAGGGTATG	CTGAGGAGGTTCAGGAATATGAG
ScpA	ATGCTGCGATCTCTCCAAATGGG	CTTGCTCGGTTACCTCACTTGTCC

## Data Availability

Data is contained within the article.
